# The impact of structured sleep schedules prior to an in-laboratory study: Individual differences in sleep and circadian timing

**DOI:** 10.1371/journal.pone.0236566

**Published:** 2020-08-12

**Authors:** William R. McMahon, Suzanne Ftouni, Andrew J. K. Phillips, Caroline Beatty, Steven W. Lockley, Shanthakumar M. W. Rajaratnam, Paul Maruff, Sean P. A. Drummond, Clare Anderson

**Affiliations:** 1 School of Psychological Sciences and Turner Institute for Brain and Mental Health, Monash University, Melbourne, Victoria, Australia; 2 Cooperative Research Centre for Alertness, Safety and Productivity, Melbourne, Victoria, Australia; 3 Cogstate Ltd., Melbourne, Victoria, Australia; 4 The Florey Institute of Neuroscience and Mental Health, The University of Melbourne, Melbourne, Victoria, Australia; University of Rome Tor Vergata, ITALY

## Abstract

**Introduction:**

Many sleep and circadian studies require participants to adhere to structured sleep-wake schedules designed to stabilize sleep outcomes and circadian phase prior to in-laboratory testing. The effectiveness of this approach has not been rigorously evaluated, however. We therefore investigated the differences between participants’ unstructured and structured sleep over a three-week interval.

**Methods:**

Twenty-three healthy young adults completed three weeks of sleep monitoring, including one week of unstructured sleep and two weeks of structured sleep with consistent bed and wake times. Circadian phase was assessed via salivary dim light melatonin onset (DLMO) during both the unstructured and structured sleep episodes.

**Results:**

Compared to their unstructured sleep schedule, participants’ bed- and wake times were significantly earlier in their structured sleep, by 34 ± 44 mins (M ± SD) and 44 ± 41 mins, respectively. During structured sleep, circadian phase was earlier in 65% of participants (40 ± 32 mins) and was later in 35% (41 ± 25 mins) compared to unstructured sleep but did not change at the group level. While structured sleep reduced night-to-night variability in sleep timing and sleep duration, and improved the alignment (phase angle) between sleep onset and circadian phase in the most poorly aligned individuals (DLMO < 1h or > 3h before sleep onset time; 25% of our sample), sleep duration and quality were unchanged.

**Conclusion:**

Our results show adherence to a structured sleep schedule results in more regular sleep timing, and improved alignment between sleep and circadian timing for those individuals who previously had poorer alignment. Our findings support the use of structured sleep schedules prior to in-laboratory sleep and circadian studies to stabilize sleep and circadian timing in healthy volunteers.

## Introduction

Numerous behavioral factors adversely impact sleep quality or quantity. Collectively referred to as poor sleep hygiene, these adverse behaviors include irregular sleep timing, exposure to light prior to bed, daytime napping, and consumption of stimulants. To minimize the impact of these factors, participants in sleep and circadian research studies are often required to adhere to a structured sleep schedule for 1–3 weeks before an in-laboratory protocol. During this time, they are further prohibited from daytime napping and the consumption of alcohol and caffeine. A structured sleep schedule will typically allow participants an 8h sleep opportunity between times that are either selected by the participant (e.g., [1, 2]), assigned to them based on their normal schedule (e.g., [3]), or identical for all participants (e.g., [4]). These pre-laboratory procedures are intended to stabilize participants’ circadian timing, satiate sleep need, and homogenize participants’ sleep outcomes prior to interventions.

The timing and duration of sleep are regulated by circadian and sleep homeostatic factors [5]. Sleep is more difficult to initiate, and of reduced duration and quality, when its timing is not optimally aligned with the circadian drive for sleep [6, 7]. For instance, awakenings are more frequent and the total duration of sleep is reduced in sleep episodes initiated during the circadian day, when endogenous melatonin levels are low [8]. Even relatively minor misalignment between the circadian clock and sleep/wake timing can result in poorer quality sleep, especially if sleep initiation occurs close to, or before, the evening secretion of melatonin, during the wake maintenance zone [9, 10]. Moreover, light is the primary time cue that resets the circadian pacemaker [11], and the timing of light exposure relative to circadian phase determines whether it will advance or delay the circadian rhythm [12, 13]. A more irregular sleep schedule pattern will lead to greater day-to-day changes in the timing and duration of light exposure and therefore greater instability in circadian timing on a day-to-day basis [14]. Theoretically, structured sleep would provide a more stable circadian phase and provide for an adequate sleep opportunity aligned with their circadian timing, but circadian phase is rarely assessed pre-study.

Beyond optimizing circadian timing, the time in bed permitted by a pre-study structured sleep schedule attempts to minimize chronic sleep deficiency prior to a laboratory study. This is especially important for studies involving sleep deprivation, where existing sleep deficiency impairs tolerance of subsequent acute and chronic sleep loss [15, 16]. Consensus statements from the American Academy of Sleep Medicine and Sleep Research Society, and the National Sleep Foundation, suggests that individuals should obtain 7–9 hours of sleep each night for optimal human health and performance [17, 18]. Indeed, in studies of healthy young adults given extended sleep opportunities of 12-16h, sleep duration eventually reaches an asymptote of 8.2–8.7h [19–21], while modeling of daily neurobehavioral responses to varying sleep opportunities suggests sleep duration should average 8.2h to maintain optimal daily performance [22]. Despite these recommendations, large individual differences exist in sleep need and sleep duration. Given that approximately one-third of young adults report inadequate sleep [23], the typical 8h sleep opportunity in a structured schedule may be a sleep extension for some participants, and yet in others result in sleep restriction; for instance, as young adults sleep between 6.5 to 7.5h when given 8h time in bed [22, 24, 25], the 8h sleep opportunity in a structured schedule may result in some participants achieving 1.7h less sleep per night than the proposed cut-off of 8.2h [22] when they enter the laboratory.

We therefore sought to test the impact of a structured 8:16h sleep:wake schedule on light exposure, circadian phase, and the duration, quality and timing of sleep, relative to an habitual unstructured sleep schedule, at both the group and individual level.

## Materials and methods

### Participants

Twenty-three healthy adults (20–43 years, 25.43±5.67 (*M±SD*); 18 men, 5 women) participated in the study. Eligible participants were fluent in English and had not travelled across three or more time zones within the past month or undertaken any shift work (defined as five or more hours worked between 10pm and 7am) within the last three months. They had a Body Mass Index between 18.0 and 29.9kg/m^2^, no history of medical, psychiatric or sleep disorders, no reported use of illicit drugs within the last year, and no consumption of caffeine exceeding 300mg/day or alcohol exceeding 14 standard units/week. Women were not currently pregnant or using hormonal contraception. All women were studied across the menstrual cycle for the four-week study, although this was controlled between participants: The three-week unstructured and structured sleep schedule in the pre-admission assessment occurred during the ovulatory, luteal and menstrual phases, while the in-laboratory study was during the follicular phase of their menstrual cycle, determined by their self-reported menses onset. Participants were in good medical and psychological health, confirmed by a full medical history and examination by a physician, including electrocardiogram and blood and urine tests, and an interview with a registered psychologist. They did not consume alcohol, caffeine, nicotine, supplements, or prescription or non-prescription drugs throughout the study, verified by a urine toxicology screen and breathalyzer assessment upon arrival to the laboratory. During screening, all participants reported habitual bedtimes between 21:30h and 01:00h, habitual wake times between 05:30h and 09:00h, and sleep duration between 7-9h with no more than one nap per week. None self-reported extreme chronotypes: Their scores ranged between 33–45 on the Morningness-Eveningness Questionnaire, indicating moderate evening or intermediate typing. Participants’ subjective sleep quality was also good: All bar one scored at or below the cut-off of 5 on the PSQI [26]; the one exception scored a 6 but met all other inclusion criteria. All participants gave written informed consent, and the Monash University Human Research Ethics Committee approved all procedures (approval number CF14/2790–2014001546).

### Pre-laboratory protocol

#### Sleep monitoring

**Fig 1:**
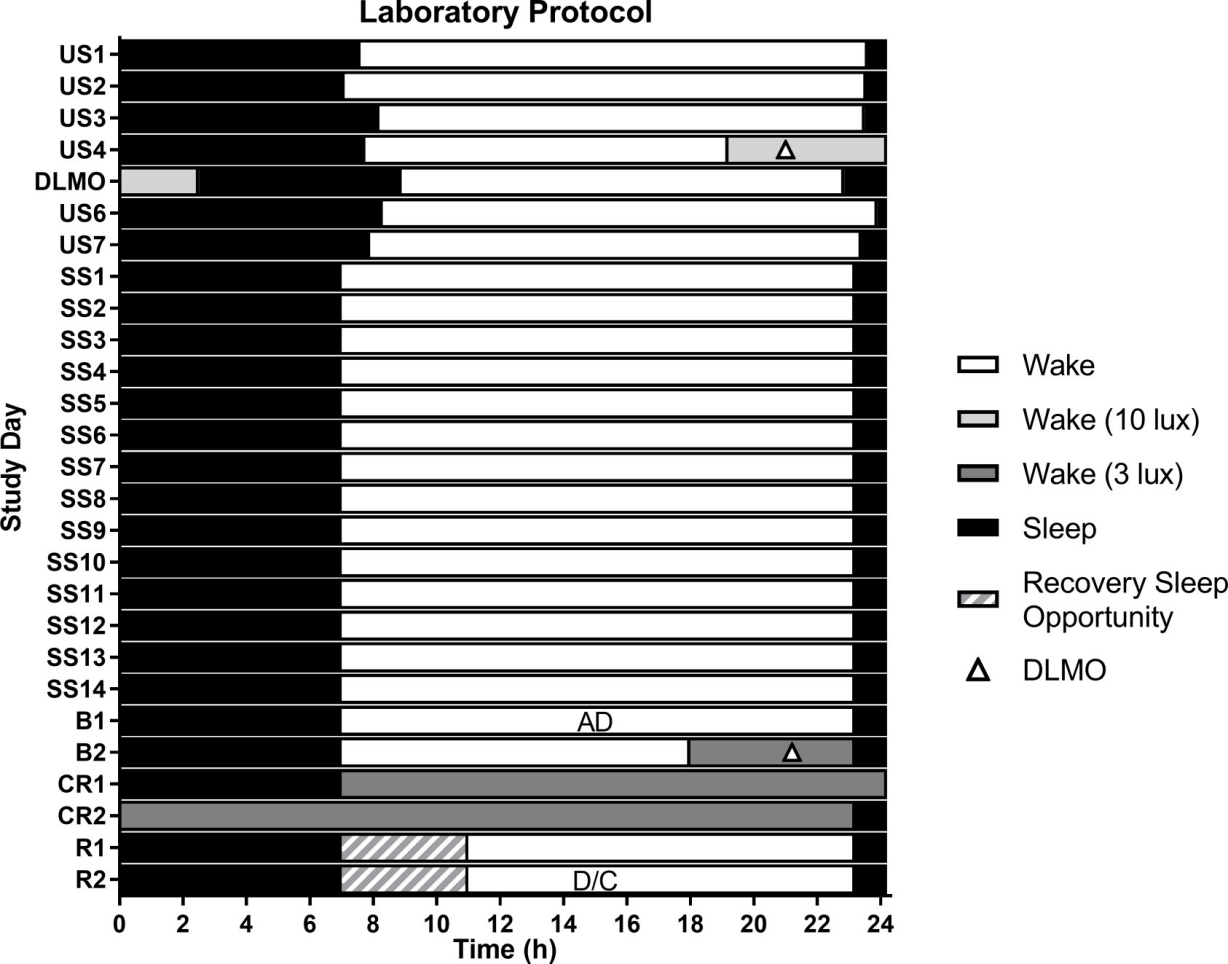
Raster plot of the protocol. Mean participant schedule shown. US, Unstructured Sleep; DLMO, at-home DLMO assessment; SS, Structured Sleep; B, Baseline; CR, Constant Routine; R, Recovery; AD, Admission; D/C, Discharge. Triangles indicate evening DLMO assessments.

For three weeks prior to the laboratory assessment, each participant wore an Actiwatch Spectrum (Philips Respironics, BMedical, QLD, Australia), completed a daily sleep diary, and made a time-stamped call-in message when they woke and when they went to bed. For the first week, participants were asked to sleep and wake at times broadly consistent with their normal schedule, but were ultimately free to sleep and wake at any time of their choosing (‘Unstructured Sleep’). For the subsequent two weeks leading into the laboratory assessment, participants adhered to a strict 8:16h sleep:wake schedule, where they maintained consistent self-selected bed- and wake times with no more than ±15min deviation (‘Structured Sleep’; see Fig 1). Participants were instructed to select a bedtime between 22:00h and 01:00h that would permit them an 8h sleep opportunity that was compatible with their other obligations (e.g., early starts for work or classes). Compliance with the sleep schedule was verified by actigraphy and time-stamped voicemail call-ins.

#### At-home circadian phase assessment

After three to four nights of Unstructured Sleep, participants completed a Dim Light Melatonin Onset (DLMO) assessment in their homes on a Friday night. This ensured all available weeknights during Unstructured Sleep were recorded prior to the DLMO assessment. Saliva samples were taken hourly, beginning 5h prior to and ending 2h after participants’ average bedtime, as calculated from sleep and wake time call-ins during the Unstructured Sleep period to that point. Participants remained awake throughout the assessment. Prior to each sample, participants were instructed to remain seated without eating for 30min and without drinking for 20min. They collected their own 2mL saliva samples via passive drool, then stored them in a domestic freezer until the beginning of the laboratory protocol. Light levels were set below 10 lux at the beginning of the assessment, as measured by a UPRTek MK350N Spectrometer at the participant’s eye level, with any windows covered by curtains. Any device with a screen that the participant intended on using was also set to below 10 lux. Participants were instructed to remain in the room under these lighting conditions except when using the bathroom, for which they were asked to wear dark sunglasses. At-home DLMOs have previously been shown to be consistent with in-lab DLMOs for a majority of participants [27, 28].

### Laboratory protocol

#### General conditions

Participants completed a six-day laboratory protocol as part of a larger study. Only the laboratory DLMO assessment is described here; the protocol is described more fully elsewhere [29, 30]. Participants were studied in a private, sound-attenuated suite free of time cues for six days: two baseline days, a 40h sleep deprivation protocol under constant routine (CR) conditions, and two recovery days. Participants maintained their Structured Sleep sleep-wake schedule during baseline, and completed a second DLMO assessment on the second baseline night. Throughout the study, temperature was maintained at 21°C ± 2°C. Illuminance levels (J17 Lumacolor photometer, Tektronix, Beavertown, USA) were measured daily in four locations around the suite directly under light panels, and were found to average ~102 ± 37 lux (horizontal plane) and ~45 ± 21 lux (vertical plane) during the baseline days, and ~3 ± 1 lux (horizontal) and ~1 ± 3 lux (vertical) during DLMO assessment. Lights were switched off during scheduled sleep.

During the laboratory DLMO assessment, saliva samples were collected hourly via passive drool from five hours prior to participants’ scheduled bedtime. The collection followed the same procedure as the at-home assessment, except that participants were not kept awake past scheduled bedtime, to avoid restricting their sleep prior to the sleep deprivation protocol that began the following day. Participants were monitored by staff for compliance with sampling procedures. Staff also collected samples and placed them in a -20°C freezer for storage.

#### Melatonin measurement

Salivary melatonin concentration was determined at the Adelaide Research Assay Facility by double antibody radioimmunoassay, using standards and reagents supplied by Buhlmann Laboratories (RKDSM-2, Buhlmann Laboratories AG, Schönenbuch, Switzerland). This assay is based on the Kennaway G280 anti-melatonin antibody [31, 32] and [125I]2-iodomelatonin as the radioligand and follows the protocol provided by Buhlmann. The minimum detection limit of the assay was 4.3pM. Samples were sent in two batches, though samples from the same participant were assayed in one batch only (i.e., samples from the same participant were not split between the two batches). Saliva samples were assayed in duplicate 200μL aliquots. The mean intra-assay coefficient of variation of the assays ranged between 7.2% and 9.4%. The range of the inter-assay coefficients of variation of the low concentration quality control was 7.2–11.5%; the range of the inter-assay coefficients of variation of the high concentration quality control was 9.6–14.2%.

### Data analysis

#### Data retention

Sleep diary data were collected from all (n = 23) participants. Three participants (1M, 2F) were removed from all analyses due to: not completing Unstructured Sleep monitoring (n = 1M); it not being possible to calculate their DLMO during Unstructured Sleep due to an extremely early DLMO (n = 1F); or disruptions to their Structured Sleep phase due to rescheduling their laboratory stay multiple times (n = 1F). Actigraphy data were lost for four other participants (4M) due to technical faults with the watches; Total Sleep Time (TST) was substituted from diary estimates for these participants due to the high degree of correlation between Actiwatch-recorded TST and diary TST in all other participants (*r* = .93). Substitution was not possible for other actigraphy variables, however, as these significantly differed from diary estimates (i.e., n = 20 for TST (17M, 3F), and n = 16 (13M, 3F) for other actigraphy calculations).

#### Sleep monitoring

Sleep timing in the actigraphy recordings was defined using sleep diary entries: Bedtime was defined as the time participants reported being in bed, attempting to fall asleep, and wake time was defined as the time participants reported waking in the morning. Actigraphy measures included: TST, the number of minutes between bedtime and wake time scored as sleep by the actiwatch; Sleep Onset Latency (SOL), the time in minutes the participant took to fall asleep after bedtime; Sleep Efficiency, the percentage of the time the participant spent in bed that was scored as sleep; and Wake After Sleep Onset (WASO), the number of minutes between sleep onset and wake time scored as wake. A root mean squared successive difference (RMSSD) was calculated as a measure of the night-to-night variability in sleep times and actigraphy variables [33].

Paired *t-*tests were used to compare the average value for each measure between i) Unstructured Sleep, with the exception of the DLMO assessment night as participants were required to delay their bedtime by two hours, and ii) the second week of Structured Sleep, with the exception of the final night, as n = 4 (20%) participants reported thoughts and worries about the laboratory stay had disrupted their sleep.

#### DLMO and phase angle calculation

DLMO times were calculated for each participant as the time at which the concentration of saliva melatonin first exceeded 3pg/mL, using linear interpolation between the samples immediately prior to and after the threshold. During the laboratory assessment, two participants’ melatonin levels did not reach the threshold before their scheduled bedtime. Their DLMO time was instead calculated 24h later, during the first evening in CR. DLMO times for these successive evenings in the laboratory were very highly correlated in the remaining participants (*r* = 0.96, n = 20). We calculated the phase angle between DLMO and the average bedtime for each participant for both the Unstructured and Structured Sleep, using their average bedtime across the respective included nights. DLMO has been reported to occur approximately 2h prior to bedtime, on average, in healthy young adults [1, 2, 4, 34–36]. As this is an average that does not necessarily account for individual variation in circadian period and sensitivity of the circadian system to light, we considered any phase angle within 1h of this, i.e., 1-3h, to represent sleep well-aligned with circadian timing [9].

#### Analyses

A false discovery rate (FDR) adjustment was used to control for the proportion of incorrectly rejected null hypotheses [37, 38] for the paired *t*-tests. FDR for significance was set to *p* < .05. Adjusted *p*-values (*p*_adj_) are presented for all these tests, using the FDR ‘*q*’ adjusted significance value. Cohen’s *d* was calculated as an estimate of effect size for all comparisons; effect sizes were classed as small (0.2 < *d* < 0.5), medium (0.5 < *d* < 0.8) or large (*d* > 0.8) according to Cohen’s criteria [39]. Cohen’s *d* values are reported as negative if performance deteriorated. All statistical analyses were performed using SPSS 21.0 (IBM Corp., Armonk, NY).

## Results

### Sleep monitoring outcomes

**Fig 2:**
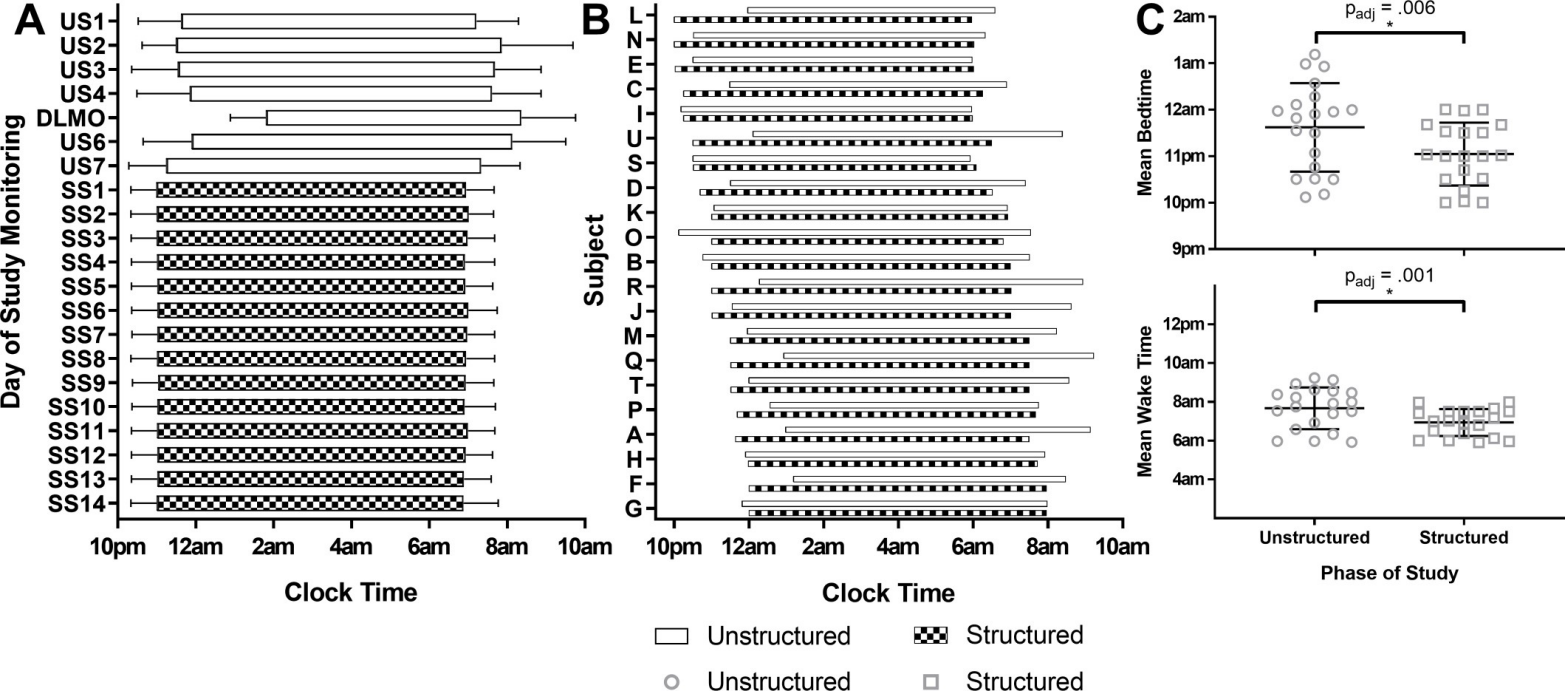
Sleep-wake timing throughout at-home sleep monitoring. **A:** Group level mean timing of each sleep period during at-home monitoring. US: Unstructured Sleep; DLMO: At-home DLMO assessment; SS: Structured Sleep. Each row shows group mean timing of one sleep episode; error bars show SD. **B:** Individual level mean timing of sleep period for each participant in Unstructured (white rectangles, top) vs. Structured (square patterned rectangles, bottom) Sleep, ordered from earliest to latest bedtime in Structured Sleep. **C:** Comparison of mean bedtime (top) and wake time (bottom) in Unstructured (circles) vs Structured (squares) Sleep. Black lines show mean and SD; symbols show individual data. *p*_adj_: False Discovery Rate adjusted *p*-value; *Statistically significant result.

The range of bedtimes was 22:07h to 01:11h (23:37 ± 57.1 mins; *M* ± *SD*) in Unstructured Sleep, and 22:00h to 00:00h (23:04 ± 40.5 mins) in Structured Sleep. During Structured Sleep, participants had significantly earlier bedtimes (by 34.5 mins on average) with a medium effect size (*t*(19) = 3.52, *p*_adj_ = 0.006, *d* = 0.79) and woke at a significantly earlier (43.9 mins on average) clock time compared to Unstructured Sleep, with a large effect size (*t*(19) = 4.78, *p*_adj_ = .001, *d* = 1.07); see Fig 2A–2C. Fig 2B highlights the individual variation in the change to the sleep timing that occurred between Unstructured and Structured Sleep: 42.9% of individuals kept largely the same sleep time (defined as change < 30 mins in either direction), while 33.3% moved sleep timing by at least 1h, and 9.5% by more than 1.5h. The same was true for wake time: Individuals either remained consistent (38.1%), or moved their wake time by more than 1 (33.3%) or 1.5h (23.8%).

**Fig 3:**
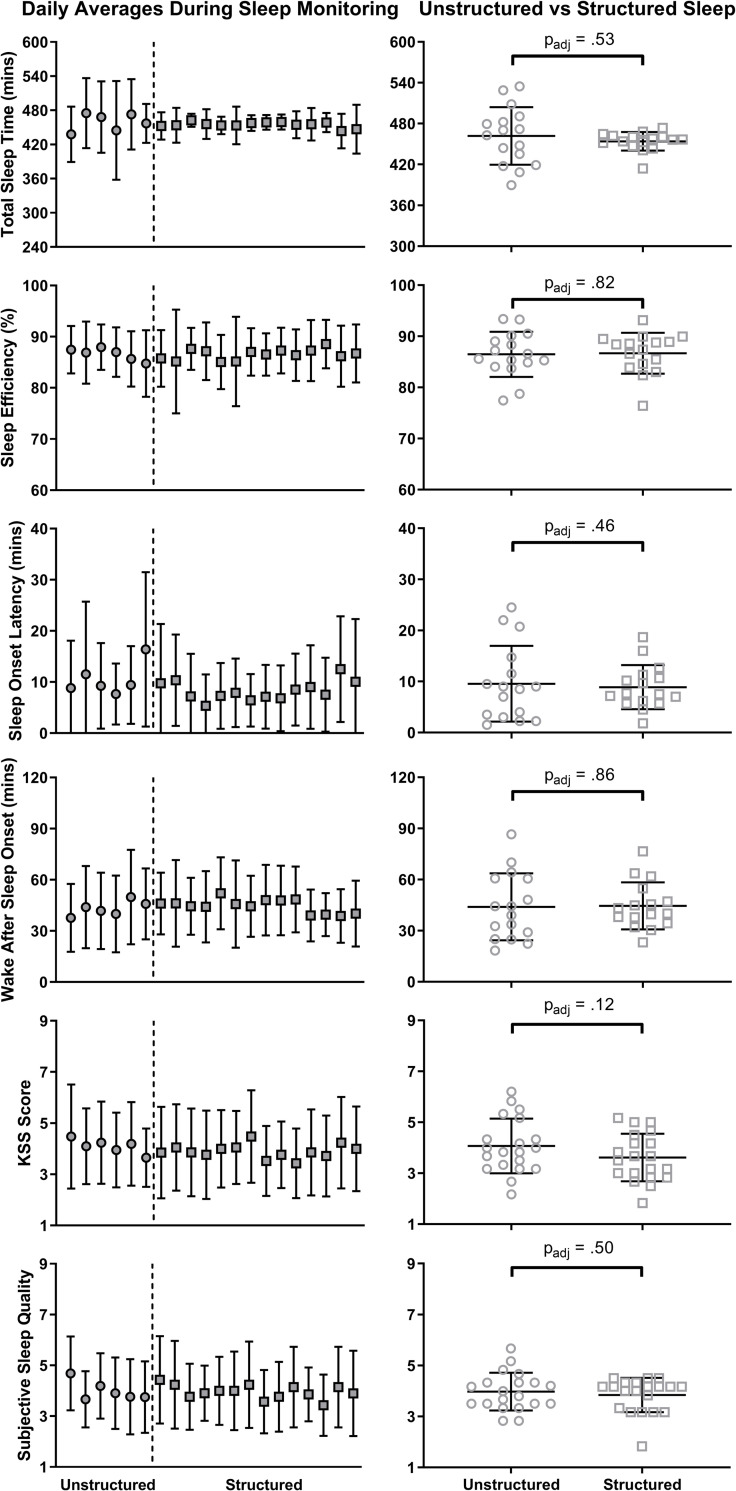
Actigraphy scored sleep outcomes in unstructured and structured sleep. Left column: Night-to-night changes in sleep outcomes (symbol = mean, error bars = SD). Unstructured Sleep: Left of vertical line, with circle symbols; Structured Sleep: right of vertical line, with square symbols. Right column: Comparison of outcomes between Unstructured (circles) and Structured (squares) Sleep. Black lines show mean and SD; symbols show individual data. *p*_adj_: False Discovery Rate adjusted *p*-value; *Statistically significant result.

No significant changes were observed at the group level for TST (*p*_adj_ = .53, *d* = 0.19), Sleep Efficiency (*p*_adj_ = .82, *d* = 0.07), SOL (*p*_adj_ = .46, *d* = 0.24), or WASO (*p*_adj_ = .86, *d* = 0.04). Subjective sleepiness upon awakening (KSS) and sleep quality also did not significantly differ between Unstructured and Structured (*p*_adj_ > .12, *d* < 0.46). Fig 3 shows these comparisons and the night-by-night changes in these variables.

At the individual level, there was a large range of TST durations (385 to 553 mins) during Unstructured Sleep. While 16/20 (80%) participants slept 7-9h (420–540 mins) during this time, this increased slightly to 19/20 (95%) participants during Structured Sleep. As expected, 13/20 (65%) participants changed by less than 30 mins in either direction from Structured to Unstructured sleep, while the remaining 7 participants either increased (3/20; 15%, average 56.6±19.1 mins) or decreased (4/20; 20%, average 59.3±23.3 mins) their TST by more than 30min. No meaningful changes were observed at the individual level for either Sleep Efficiency (-3.3% to 5.4%) or SOL (15 mins shorter to 9.7 mins longer); all individual SOL values during Structured Sleep were within MSLT criteria for normal sleep [40]. For WASO, 5/16 (31.3%) in Unstructured, and three of the same five (18.75% of the total) in Structured, had WASOs >60min. In Unstructured, 2/16 (12.5%, average 22.6±0min) improved while 2/16 (12.5%, average 20.6±3.4) deteriorated by >15min.

As expected, RMSSDs were significantly lower in Structured Sleep relative to Unstructured for bedtime (*t*(15) = 6.32, *p*_adj_ = .001, *d* = 1.39), wake time (*t*(15) = 4.24, *p*_adj_ = .003, *d* = 1.01), and TST (*t*(15) = 4.54, *p*_adj_ = .002, *d* = 1.09), indicating reduced night-to-night variability in participants’ sleep duration and timing during Structured Sleep. RMSSDs did not significantly change for SOL, Sleep Efficiency, or WASO (*p*_adj_ > .45, *d* < 0.27). Table 1 summarizes RMSSD values.

**Table 1:** Variability in sleep outcomes during unstructured and structured sleep.

		US		SS			
		*Mean*	*SD*	*Mean*	*SD*	*p*_adj_	*d*
RMSSD	Bedtime (h)	0.91	0.63	0.04	0.05	.001	1.39
	Wake time (h)	0.91	0.63	0.19	0.23	.003	1.01
	TST (min)	71.00	36.01	27.87	13.75	.002	1.09
	SOL (min)	10.82	6.68	9.04	3.67	.46	0.23
	Sleep Efficiency (%)	4.59	2.03	5.40	2.85	.45	-0.26
	WASO (min)	18.40	9.57	15.47	6.91	.45	0.27

US: Unstructured Sleep; SS: Structured Sleep; RMSSD: Root Mean Squared Successive Differences; TST: Total Sleep Time; SOL: Sleep Onset Latency; WASO: Wake After Sleep Onset; *p*_adj_: False Discovery Rate adjusted *p*-value; *d*: Cohen’s *d*

### Changes in circadian phase and circadian timing of sleep

**Fig 4:**
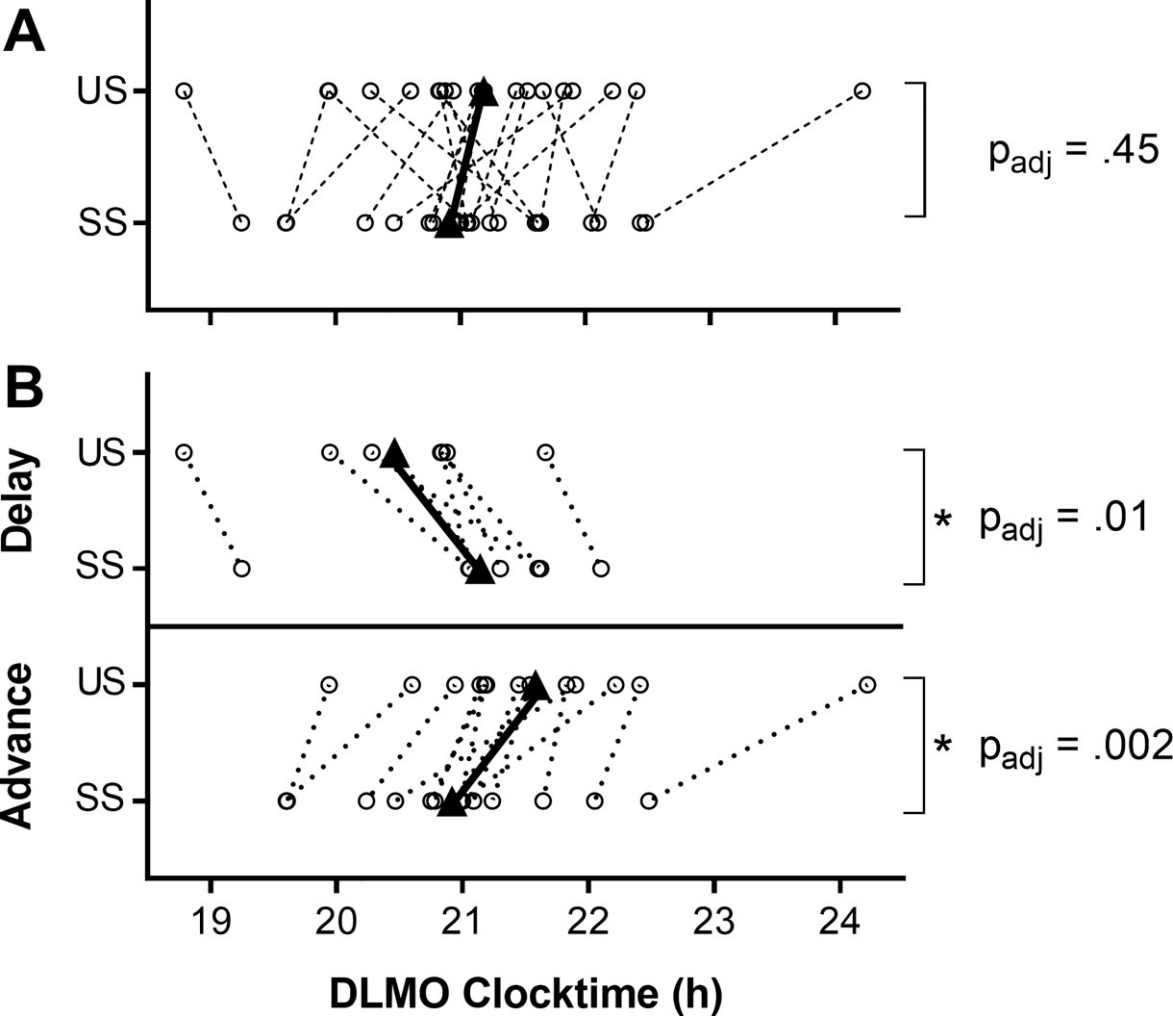
Changes in circadian phase between unstructured and structured sleep. **A**: All participants; **B**: The group split by the direction of the phase shift that occurred. US: Unstructured Sleep; SS: Structured Sleep. Black triangles joined by black line show group mean; circles show individual data. *p*_adj_: False Discovery Rate adjusted *p*-value; *Statistically significant result.

There was no change at the group level in circadian phase between Unstructured and Structured Sleep (*p*_adj_ = .45, *d* = 0.24; Fig 4 –upper panel). This is likely due to the individual variation observed: Seven participants showed a significant phase delay averaging 41 *±* 25 mins (*t*(6) = -4.40, *p*_adj_ = .01, *d* = -1.66), while 13 had a significant phase advance averaging 40 *±* 31 mins (*t*(12) = 4.65, *p*_adj_ = .002, *d* = 1.29). See Fig 4B.

**Fig 5:**
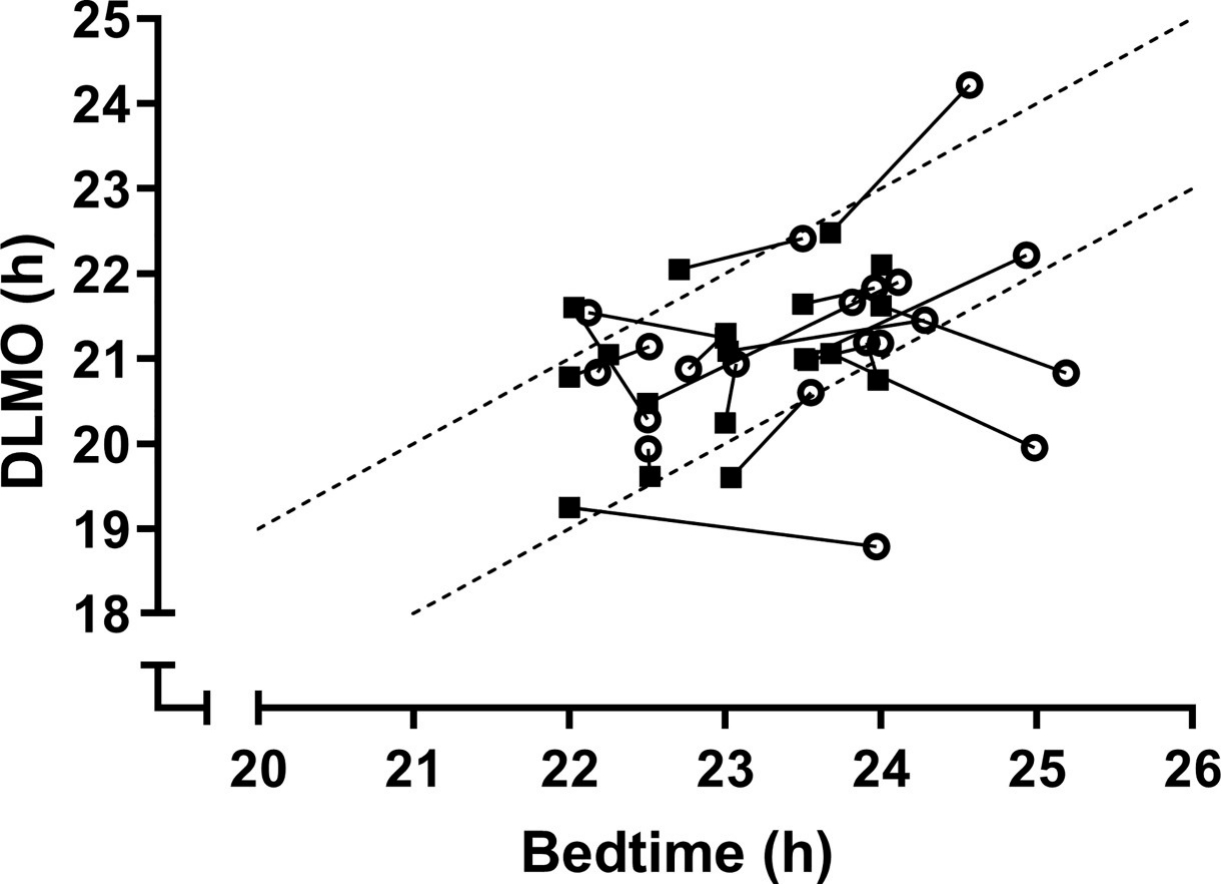
Change in phase angle between DLMO and bedtime. Unstructured Sleep (circles) and Structured Sleep (squares) Sleep; each symbol is one participant. Dashed grey lines enclose a 1-3h range for a well-aligned phase angle between DLMO and bedtime.

Phase angle between DLMO and bedtime did not significantly change between Unstructured and Structured Sleep (*p*_adj_ = .22, *d* = 0.37). As shown in Fig 5, 75% (15/20) of participants had a well-aligned phase angle of 1 to 3h during Unstructured Sleep. For those outside this range (n = 5), three participants had a phase angle > 3h, and 2/5 had a phase angle < 1h; their mean absolute deviation from the 1-3h window was 1.33*±*0.79h, with a range of 0.42–2.18h. Following two weeks of structured sleep, the number of participants in the 1-3h window increased slightly to 80% (16/20) of participants, including all participants who were outside this range during Unstructured Sleep. Of the 4/20 (20%) participants who moved outside the 1-3h phase angle range following Structured Sleep, their mean absolute deviation was minimal (0.40*±*0.14h, with a range of 0.23–2.57h), with no impact on sleep duration (*p*_adj_ = .43) or WASO (*p*_adj_ = .83).

## Discussion

Our data demonstrate that, compared to Unstructured Sleep, adherence to an 8h structured sleep schedule, a typical screening approach for many sleep and circadian in-laboratory studies, resulted in earlier bed- and wake-times, decreased variability in sleep timing and total sleep time, and shifted circadian phase (though the direction of the shift varied between individuals). No changes in other sleep parameters (WASO, SOL, sleep efficiency) or timing of sleep relative to circadian phase (phase angle) were observed at the group level; small changes were observed for some individuals.

Our data could suggest that there is little need for a structured sleep schedule in such a thoroughly screened population, given that a majority of the sample showed little change in the circadian timing of sleep (phase angle) or sleep quality and quantity between Unstructured and Structured Sleep. As seen in Fig 5, however, the Structured Sleep schedule clearly benefited individuals whose phase angle was initially outside a well-aligned range, as all these individuals subsequently were well-aligned. This suggests studies seeking to examine individual vulnerability could control for the impact of poor alignment between sleep and circadian timing on in-laboratory outcomes by either recording and accounting for individual sleep and circadian timing, or by assigning individuals a structured sleep schedule of up to two weeks’ duration (though a shorter duration may also achieve the same result). This consideration may also be more important in smaller or less rigorously screened samples: Our criteria excluded individuals who frequently napped or with sleep and wake times outside a certain range. In addition, Structured Sleep led to more homogeneity between and within individuals, particularly in sleep timing. Stability in sleep timing is important for better alignment between sleep and circadian timing, improves sleep quality, and has been linked to improved academic outcomes [41]. As a result, we conclude that the Structured Sleep protocol achieved its aims of ensuring participants were well-rested, sleeping at regular and better aligned circadian times, and that sleep outcomes were more consistent across individuals.

The at-home DLMO assessment was conducted in less controlled conditions than the laboratory assessment, which may limit their comparability. While light levels were restricted to <3 lux in the laboratory, this is impractical in at-home testing, so a higher threshold of <10 lux was used. Though still dim, 10 lux is sufficient to partially suppress the melatonin rise, potentially leading to a later estimate of DLMO. For instance, our previous work reported that DLMOs measured under 10 lux are, on average, 22min later than in <1 lux light for the same participants, with some individuals showing evidence of melatonin suppression in light illuminance as low as 6 lux [42]. It is also possible that participants did not remain in the <10 lux room for the entire duration of the at-home DLMO assessment, though they were instructed to do so unless using the bathroom, and to wear dark sunglasses if exiting the room for any reason. Actigraphy light data did not suggest these instructions were violated, and visual inspection of the obtained melatonin curves did not show evidence of substantial melatonin suppression.

Our study had limitations that should be considered when interpreting the results. First, our sample was relatively small, and consisted of healthy young adults; consequently, our results may not be generalizable to older adults, shift workers, or individuals with sleep disorders. Our sample also contained few women, due partly to our screening for use of hormonal contraception, and partly to our requirement that female participants be scheduled to complete the laboratory stay during the follicular phase of their menstrual cycle. Second, our small sample size may have meant we were not adequately powered to detect an effect in some of our comparisons. Given this, we used a less conservative method of correction for multiple comparisons, and report effect sizes for all analyses conducted. In particular, it is unlikely that we had the statistical power to detect the small phase shifts we observed with our model of light and sleep patterns. Third, a degree of compromise was required to protect the other aims of the study (published elsewhere) and to ensure participants could admit to the study: These included starting the Unstructured period later in the week for some participants, resulting in fewer nights of data prior to the at-home DLMO assessment, and curtailing the in-lab DLMO assessment at participants’ Structured bedtime to ensure they would get a full 8h sleep opportunity prior to undergoing sleep deprivation. Fourth, some of the variation observed in the Unstructured period may be attributable to the effects of catching up sleep following the at-home DLMO assessment. The pattern of results observed remains the same when these weekend days are omitted from the Unstructured data, however.

Our findings support the use of a structured sleep/wake schedule prior to a sleep and circadian study and have implications for future studies that use structured sleep schedules. First, several of our participants experienced a reduction in their TST during Structured Sleep, indicating that an 8h sleep opportunity may not be adequate for all individuals, especially young adults. A 9h sleep opportunity would be in line with recommendations for young adults [17], and has been shown to result in 7.93h sleep on average [43]. A longer sleep opportunity may, however, result in reduced sleep efficiency, especially for individuals accustomed to sleeping less than 8h habitually (62% of our sample). Structured sleep schedules should therefore give participants adequate time to sleep (a minimum 8h in young adults), but not so long that sleep quality is compromised by reduced sleep efficiency. Second, the impact of work requirements on sleep-wake schedules should be considered, as participants who need to wake early for work may have to select a sleep-wake schedule that is less suited to their circadian timing than those who do not work and thus have more freedom when selecting a sleep schedule. Third, though our sample did not include clinical patients, our results may suggest that assessing circadian phase in clinical populations will be more accurate if a consistent sleep pattern is maintained prior to the assessment; however, this may not be essential if an approximation of circadian phase is sufficient. Fourth, measuring the impact of changing light exposure patterns on circadian phase may require a device that records light closer to participants’ eyes. Controlling for other factors that can influence circadian timing, such as the period of the circadian pacemaker and non-photic zeitgebers, may also help improve predictions. Overall, though, structured sleep schedules are an effective means of ensuring participants are well-rested, well-aligned, and achieve sleep of consistent duration, timing, and quality prior to in-laboratory testing.

## Supporting information

S1 Data (XLSX)
